# Effect of respiratory rate and size of cannula on pressure and dead-space clearance during nasal high flow in patients with COPD and acute respiratory failure

**DOI:** 10.1152/japplphysiol.00769.2021

**Published:** 2022-01-27

**Authors:** Maximilian I. Pinkham, Ulrike Domanski, Karl-Josef Franke, Justus Hartmann, Maik Schroeder, Tony Williams, Georg Nilius, Stanislav Tatkov

**Affiliations:** ^1^Fisher & Paykel Healthcare Ltd., Auckland, New Zealand; ^2^Vamed Klink Hagen-Ambrock, Hagen, Germany; ^3^Märkische Kliniken GmbH, Lüdenscheid, Germany; ^4^Evang. Kliniken Essen-Mitte GmbH, Essen, Germany; ^5^Universität Witten/Herdecke, Witten, Germany; ^6^Middlemore Hospital, Auckland, New Zealand

**Keywords:** acute respiratory failure, dead space, nasal high flow, respiratory rate, work of breathing

## Abstract

Nasal high flow (NHF) is an efficient oxygenation tool for the treatment of respiratory failure. The study investigated the effect of breathing pattern on positive airway pressure and dead-space clearance by NHF. The breathing cycle during NHF was characterized in 26 patients with acute respiratory failure (ARF) and stable COPD and after mechanical ventilation (post-MV) via tracheostomy where also pressure was measured in the trachea. Dead-space clearance was measured in airway models during different breathing patterns. NHF reduced the respiratory rate (RR) and T_I_/T_E_ through prolonging the T_E_; the T_I_/T_E_ ranged between ≤0.5 observed in the COPD patients and ∼1.0 in the ARF patients. NHF via a standard medium-sized cannula interface generated a low-level expiratory pressure proportional to NHF rate and breathing flow; the median generated positive end-expiratory pressure was only 1.71 cmH_2_O at NHF 45 L/min. The dilution and purging of expired gas from a nasal cavity model were observed to occur at the end of expiration as expiratory flow slowed and the dynamic pressure decreased. The higher RR with shorter end-expiratory period resulted in reduced dead-space clearance by NHF; 20 L/min cleared 43 ± 2 mL at RR 15 min^−1^ vs. 9 ± 5 mL at RR 45 min^−1^, *P* < 0.001, which was increased at higher NHF rate. At lower RR, the clearance was similar between NHF rates 20 and 60 L/min. Higher NHF rates elevate positive airway pressure, and at the increased RR can improve the clearance. This may enhance gas exchange and lead to a reduction in the work of breathing.

**NEW & NOTEWORTHY** During nasal high flow (NHF) an increased breathing frequency, which is commonly observed in acute respiratory failure, can lead to decreased dead-space clearance. Higher NHF rates increase the clearance and reduce the rebreathing which may eventually lower the respiratory rate and the work of breathing. Monitoring of the respiratory rate could be an important indicator of not only the respiratory function but also the NHF rate selection and the therapy efficacy.

## Graphical abstract

**Figure:**
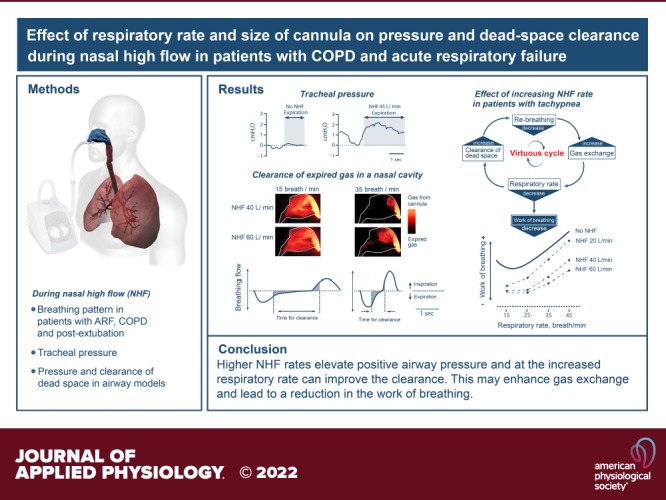

## INTRODUCTION

Nasal high flow (NHF) therapy delivers a constant flow of heated and humidified gas, usually 20 to 60 L/min in adults, via nasal cannula. NHF with supplemental oxygen is an efficient oxygenation tool that provides respiratory support used in the treatment of a wide range of patients with respiratory failure, including COVID-19 (1, 2).

Pressure is a product of flow and resistance, and during NHF the generated airway pressure is dependent on the selected flow rate and level of occlusion of the nares (3, 4). Nare sizes can vary significantly, which may cause variability in the resistance that is generated by the NHF cannula and therefore the generated pressure. The open system of the NHF interface makes the measurement of the airway pressure difficult, and it is not typically performed outside of a research setting. The cause of the variability in the generated pressure during NHF requires further clarification.

NHF is delivered using loosely fitting nasal cannula that can dilute and purge the expired gas from the anatomical dead space via the leak around the prongs (5, 6). A reduction of rebreathing may enhance the oxygenation efficiency, lead to a decrease in carbon dioxide (CO_2_) retention, and reduce the work of breathing (1, 2, 7, 8). The volume of expired gas that is cleared from the upper airways is flow and time dependent (5, 6), and the available time for clearance may be affected by the breathing cycle. However, the breathing pattern during NHF therapy and across different patient populations in relation to dead-space clearance has not been well defined. A reduction of the RR in response to NHF has been reported in numerous studies (2, 9–14). An understanding of how the RR relates to the dead-space clearance and the subsequent physiological effects may have important implications for the application of the NHF therapy.

The study aimed to characterize how the breathing cycle may affect the dead-space clearance by NHF. Furthermore, the authors hypothesized that differences in the nare size and breathing pattern may explain the variability in the generated airway pressure that is observed during NHF.

## MATERIALS AND METHODS

### NHF in Stable COPD Patients

Nine stable patients with chronic obstructive pulmonary disease (COPD) were recruited during their control visit in the pneumological ward, which was approved by the Ethics Committee of Witten-Herdecke University, Germany, and registered under https://clinicaltrials.gov (NCT05053074); written informed consent was obtained from each participant. Five patients required long-term oxygen treatment, and three patients had chronic hypercapnia with Pco_2_ values of >50 mmHg: see Table 1 for baseline characteristics. During the visit, 1 h of no NHF was followed by 1 h of NHF at 30 L/min and the patient remained awake. The flow rate of 30 L/min was selected based on previous experience that some stable patients may not tolerate the higher flows and to ensure consistent data over the full 1 h of recording. The NHF was generated by the AIRVO 2 (Fisher & Paykel Healthcare, New Zealand) and delivered via nasal cannula (OPT944+, Fisher & Paykel Healthcare, New Zealand). Respiration was recorded and transcutaneous CO_2_ and SpO_2_ were continuously monitored (Sentec, Switzerland).

**Table 1.** Baseline characteristics of the patients with stable chronic obstructive pulmonary disease

Patient Characteristics	
*n*	9
Age, yr	68 ± 4
Height, cm	172 ± 9
Weight, kg	65 ± 14
Supplemental O_2_ required, no. (%)	5 (56)
Supplemental O_2_, L/min (FIO_2_)	1.6 ± 0.4
Pco_2_, mmHg	47.3 ± 5.7
pH	7.4 ± 0.0
FEV_1_, %predicted	28 ± 11
FVC, %predicted	52 ± 17
TLC, %predicted	119 ± 17
RV, %predicted	225 ± 40
dl_CO_, %predicted	32 ± 20

Data are means ± SD. FEV_1_, forced expiratory volume in 1 s; FVC, forced vital capacity; TLC, total lung capacity; RV, residual volume; Dl_CO_, lung diffusing capacity for carbon monoxide.

### NHF in Tracheostomized Patients Recently Weaned from Mechanical Ventilation

Ten recovering patients were recruited, who had all recently been successfully weaned from long-term mechanical ventilation (MV) via a stoma in trachea but capable of maintaining unassisted nasal breathing. Two patients were not included in the analysis due to incomplete data (see Table 2 for the patient baseline characteristics). The study was approved by the Ethics Committee of Witten-Herdecke University, Germany, and registered under https://clinicaltrials.gov (NCT01509703); written informed consent was obtained from each participant. A period of recording was obtained during no NHF (i.e., baseline) followed by 5 to 10 min each of NHF at 15 L/min, 30 L/min and 45 L/min. The period of recording per intervention was adjusted to obtain values of the airway pressure with the absence of artefact over a sufficient number of breathing cycles. The NHF was generated by the AIRVO 2 (Fisher & Paykel Healthcare, New Zealand) and delivered via nasal cannula (OPT944+, Fisher & Paykel Healthcare, New Zealand).

**Table 2.** Baseline characteristics of the patients recovering in the respiratory ward following mechanical ventilation and in the ICU/HDU with acute respiratory failure

Patient Group	Post-MV	ARF
*n*	8	9
Age, yr	69 ± 8	57 ± 18
Height, cm	171 ± 7	165 ± 6
Weight, kg	72 ± 11	93 ± 22
Supplemental O_2_ required, no. (%)	5 (63)	9 (100)
Supplemental O_2_, L/min (FIO_2_)	2.1 ± 2.0	(0.36 ± 0.11)
Pco_2_, mmHg	43.5 ± 7.0	
pH	7.4 ± 0.0	
Primary cause of hospitalization		
COPD exacerbation, no. (%)	3 (37.5)	
Neurologic disease (including stroke), no. (%)	2 (25)	
Pneumonia, no. (%)	2 (25)	4 (45)
Sepsis, no. (%)		3 (33)
Other, no. (%)	1 (12.5)	2 (22)

Data are means ± SD; post-MV, following minute ventilation; ARF, acute respiratory failure; COPD, chronic obstructive pulmonary disease; ICU/HDU, Intensive Care Unit/High-Density Unit.

### NHF in Patients with Acute Respiratory Failure

This part of the study was approved by the Health and Disabilities Ethics Committee, New Zealand, and registered under http:/anzctr.or.au/ACTRN12619000831189. Nine patients with ARF in the Intensive Care Unit/High-Dependency Unit of Middlemore Hospital, Auckland, New Zealand, were included in the study, and written informed consent obtained (see Table 2). The breathing data were continuously measured for 30 min while each participant received NHF as per the standard hospital protocol. The NHF was generated from pressurized air wall source, heated to 37°C, and humidified to 100% relative humidity by the F&P 950 (Fisher & Paykel Healthcare, New Zealand) and delivered via nasal cannula (OPT944+, Fisher & Paykel Healthcare, New Zealand).

### Breathing Data and Airway Pressure

Respiratory inductance plethysmography (RIP) (Respitrace QDC; Viasys Services) was used to record breathing, as described previously (15, 16). In the postextubated patients, a probe was placed through the tracheostomy retainer to measure the airway pressure (6). The RIP signal and the pressure recordings were synchronized to determine the airway pressure throughout the breathing cycle. In the patients recently weaned from MV, the RIP signal was calibrated to measure tidal volume and minute ventilation (15, 16).

### NHF in Airway Models

In the bench model, the NHF was generated by pressurized wall source (600 KPa) and delivered via a medium-sized nasal cannula interface with an inner diameter (ID) of 5.42 mm and outer diameter (OD) of 6.22 mm (OPT944+, Fisher & Paykel Healthcare, New Zealand). The upper airway model was three-dimensionally printed and was based on the averaged geometry of people from the same European population (17). CAD software was used to reduce the cross-sectional area of the nares (original cross-sectional area of nares; left: 115.9 mm^2^/right: 117.5 mm^2^) in 10% increments down to 50% of the original area. Airway pressure was measured in the static condition without simulated breathing flow (PA-1 Pneumotach Amplifier, series 1110, Hans Rudolph Inc.). The combined effect of the NHF rate and cross-sectional area of the model nares on generated pressure was fit using *Eq. 1*: pressure = *k* × area*^a^*, where *k* is the *Y*-intercept at *X* = 0 and *a* is the slope of the log pressure × log cross-sectional area plot. The end-expiratory pressure that was measured in the recovering patients’ post-MV was used to estimate the total cross-sectional area of the nares with the rearrangement of *Eq. 1*: = ${\left(\frac{\text{PEEP}}{k}\right)}^{1/a}$, where PEEP is positive end-expiratory pressure.

The rate of dead-space clearance by NHF was quantified in the full upper airway model during zero breathing flow. A chamber (∼5 cm^3^) created a seal around the nares while the nasal cannula was in place. All gas exiting the nares was passed through an optical capnograph (OG-3800K and TG-980P, Nihon Kohden, Japan). The upper airway model was filled with carbogen gas (6% CO_2_-20.9% O_2_-73.1% N_2_), and then the NHF of room air (0.04% CO_2_) was applied instantaneously by switching a valve that was placed between the pressurized wall source and airway model to redirect the flow through the cannula. The protocol was completed six times per NHF rate. One-phase decay curves were fitted to the data (*R*^2^ > 0.99), and the half-life (*t*_1/2_) was calculated. The NHF was delivered via the medium-sized nasal cannula and smaller nasal cannula with ID of 3.4 mm and OD of 4.2 mm (OPT416, Fisher & Paykel Healthcare, New Zealand).

**Figure 1.**
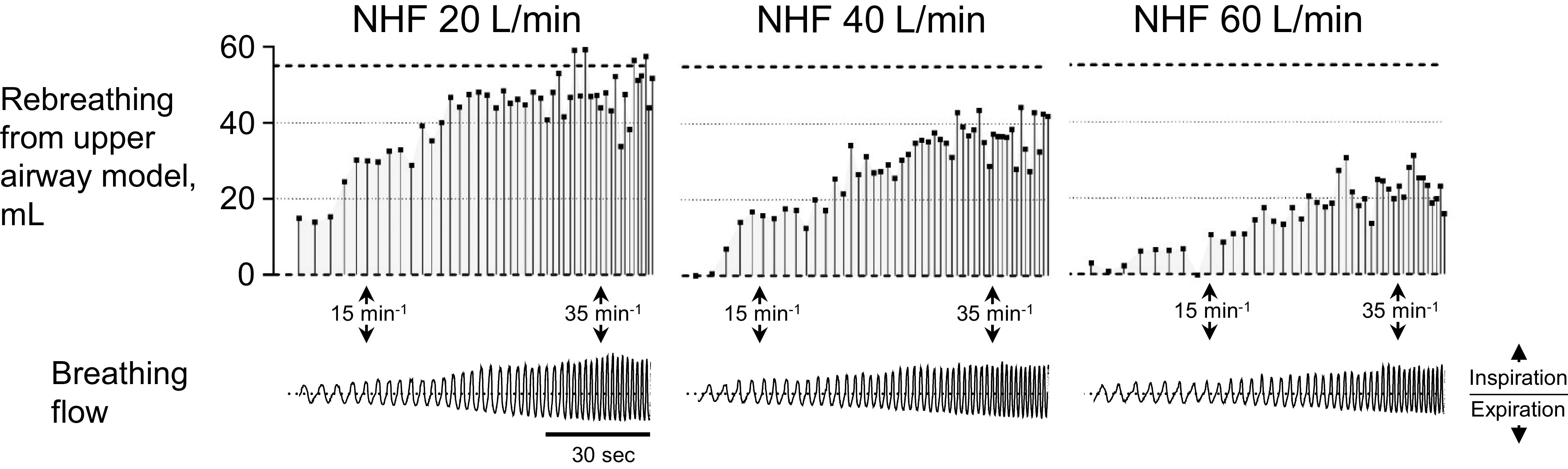
The graphs show the breath-to-breath variability of the rebreathing from an upper airway model during nasal high flow (NHF) at 20 L/min, 40 L/min, and 60 L/min: please refer to the materials and methods for a full description of the experimental set-up. Data are presented per breath.

The rebreathing of expired gas during NHF was quantified by volumetric capnography using a low-resistance pneumotachograph (Fleisch, Switzerland) and optical capnograph (OG-3800K and TG-980P, Nihon Kohden, Japan) that were placed in line with the upper airway model. Pilot testing on the rebreathing during variable breathing pattern was performed while an operator breathed through a mouthpiece filter attached to a full upper airway model (5) for ∼2 min. The results indicated that the rebreathing during NHF can vary breath-to-breath (Fig. 1). To produce a uniform breathing waveform, the lung simulator was programmed to reflect the patient data of the current study (Supplemental Fig. S1; see https://doi.org/10.6084/m9.figshare.16993051.v3). The RR was set at 5, 15, 25, 35, and 45 min^−1^, and the tidal volume was adjusted according to the RR; 550 mL, 500 mL, 450 mL, 400 mL, and 350 mL, respectively. CO_2_ was entrained directly into the simulator to maintain an end-tidal CO_2_ of ∼5%.

To observe gas kinetics in upper airway, CO_2_ was visualized using infrared spectroscopy (SC7600, Teledyne FLIR LLC) (5) in a model of a nasal cavity that was machined in an aluminum block, coated with Vantablack (Surrey NanoSystems, UK), covered by a sapphire optical window and heated to 160°C (320°F). For this part of the study, the NHF was generated by the AIRVO 2 to enable the entrainment of a carbogen gas mix (6% CO_2_-21% O_2_-73% N_2_) via the inlet oxygen to visualize the gas from the cannula. The breathing flows were generated by a lung simulator (ASL 5000, IngMar Medical) and were halved to account for the single nasal cavity.

### Modeling of Work of Breathing

The effects of dead-space clearance on the work of breathing was modeled based on Otis et al. (18) The equation that was used to calculate the work per minute was $5,000f\;{\left(\frac{\dot{V}A}{f}+VD\right)}^{2}+150{\left(\dot{V}A+VDf\right)}^{2}+3{\left(\dot{V}A+VDf\right)}^{3}$ where *f* is breathing frequency, $\dot{V}A$ is alveolar ventilation, and *VD* is dead-space volume per breath. The constants provide for the effort required to overcome the elastance and resistance in healthy man with high lung compliance (18). For the purpose of the current study, a constant $\dot{V}A$ of 4 L/min, *VD* of 0.2 L, and a sinusoidal breathing pattern were assumed. The work that is required to overcome the elastance is indicated by $5,000f\;{\left(\frac{\dot{V}A}{f}+VD\right)}^{2}$ and is greater with increasing tidal volume. The work that is required to overcome the viscous forces is provided by $150{\left(\dot{V}A+VDf\right)}^{2}+3{\left(\dot{V}A+VDf\right)}^{3}$ and is predominantly affected by changes in the dead-space ventilation that is given by *VD* × *f*. The cleared volume of dead space by NHF that were determined from the bench experiments were subtracted from the *VD*. The effect of reduced lung compliance was presented by multiplying the elastance constant by 4×.

### Data Analysis

The RIP signal was analyzed to obtain inspiratory time (T_I_), expiratory time (T_E_), respiratory cycle time (T_Tot_), and RR. In the COPD patients, 50 breaths per patient with and without NHF were compared using a paired *t* test. In the patients recently weaned from mechanical ventilation, 20 breaths per patient during no NHF and during the NHF rates of 15 L/min, 30 L/min and 45 L/min was compared with repeated measures One-Way ANOVA with Tukey’s post hoc. In the group of patients with ARF, all breaths were analyzed that were unaffected by artifact (e.g., body movement). The best-fit line of the relationship between the T_E_ and RR was determined using log T_E_ × log RR plot. The pressure data in patients was analyzed using the Friedman test with Dunn’s multiple comparisons. The bench data were analyzed using a One-Way ANOVA with Tukey’s post-hoc test. The analog signals were digitized using a 16-bit ADC (ADI PowerLab 16/30; ADInstruments, New Zealand), recorded at 1,000 Hz and then analyzed using LabChart V8 software. The statistical analysis was performed using GraphPad Prism V5.01 (GraphPad Software) and the threshold for statistical significance was set at *P* < 0.05. Data are presented as means ± SD unless stated otherwise.

## RESULTS

### Breathing Pattern Response to NHF

In stable patients with COPD, NHF at 30 L/min lengthened the T_E_ and decreased the RR and T_I_/T_E_ (Table 3). In the group of patients who were successfully weaned from MV and in the respiratory ward, the application of NHF resulted in a flow-dependent reduction in the RR and T_I_/T_E_ (Table 4).

**Table 3.** Breathing parameters during no NHF and NHF at 30 L/min in chronic obstructive pulmonary disease patients

	No NHF	NHF 30 L/min
*n*	9	9
Respiratory rate, min^−1^	18 ± 4	13 ± 4*
Tidal volume, mL	390 ± 130	520 ± 220
Minute ventilation, L/min	6.88 ± 3.28	6.23 ± 1.88
T_I_, s	1.17 ± 0.36	1.34 ± 0.49
T_E_, s	2.46 ± 0.62	3.80 ± 1.47*
T_I_:T_E_ ratio	0.51 ± 0.12	0.39 ± 0.06*
TcCO_2_, mmHg	46.1 ± 7.6	46.2 ± 5.7
SpO_2_, %	97 ± 5	97 ± 4
Received supplemental O_2_, no.	6	5

Data are means ± SD; T_I_, inspiratory time; T_E_, expiratory time; TcCO_2_, transcutaneous partial pressure of carbon dioxide. *P < 0.05, significant difference between no nasal high flow (NHF) and NHF at 30 L/min.

**Table 4.** Breathing parameters and positive airway pressure recorded during no NHF and NHF at 15 L/min, 30 L/min, and 45 L/min in tracheostomized patients

	No NHF	15 L/Min	30 L/Min	45 L/Min
*n*	8	8	8	8
Respiratory rate, min^−1^	18 ± 5	16 ± 3	14 ± 4	14 ± 4
Tidal volume, mL	410 ± 170	420 ± 170	460 ± 220	450 ± 210
Minute ventilation, L/min	6.8 ± 2.8	6.2 ± 2.2	6.4 ± 3.5	5.7 ± 1.7
T_I_, s	1.65 ± 0.70	1.64 ± 0.49	1.59 ± 0.35	1.56 ± 0.45
T_E_, s	2.28 ± 0.92	2.51 ± 0.67	2.75 ± 0.67	3.29 ± 1.01
T_I_:T_E_ ratio	0.75 ± 0.14	0.68 ± 0.18	0.62 ± 0.15	0.53 ± 0.17*†
Airway pressure median				
PEEP, cmH_2_O	0.12 (0.00, 1.28)	0.66 (0.17, 1.10)	1.00 (0.31, 2.96)	1.71 (0.71, 7.06)
PIP, cmH_2_O	−1.87 (−3.78, −0.20)	−1.70 (−3.35, −0.60)	−1.75 (−3.48, −0.45)	−0.87 (−2.30, −0.07)
PEP, cmH_2_O	2.06 (−0.07, 3.69)	2.03 (1.12, 4.52)	2.69 (1.35, 6.74)	4.13 (1.58, 9.81)*†

Data are means ± SD; T_I_, inspiratory time; T_E_, expiratory time; PEEP, positive end expiratory pressure; PIP, peak inspiratory pressure; PEP, peak expiratory pressure. *P < 0.05, significant difference compared with no nasal high flow (NHF); †*P* < 0.05, significant difference between no nasal high flow (NHF) and NHF at 15 L/min.

### Characterizing the Breathing Pattern during NHF

**Figure 2.**
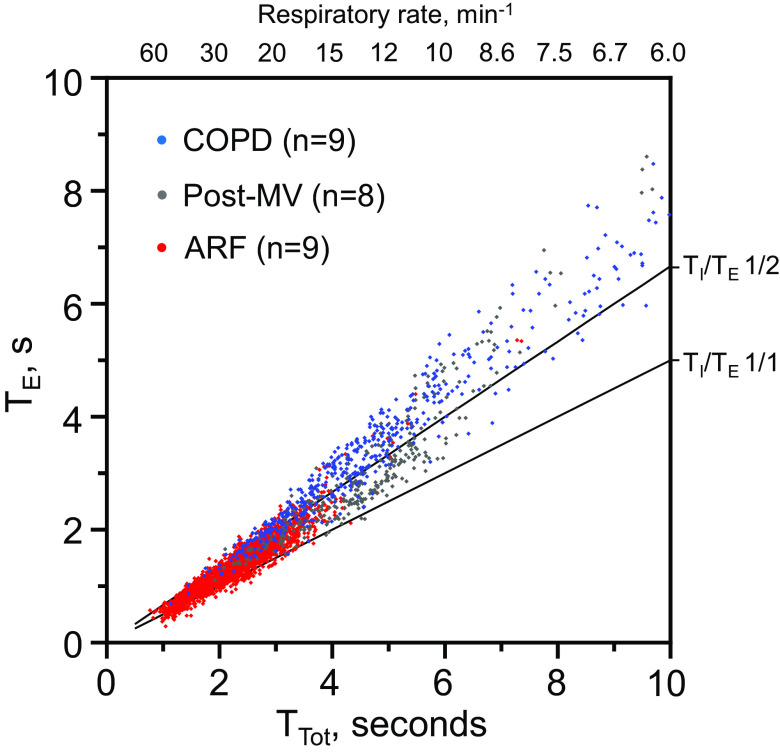
Per-breath data on the expiratory time (T_E_) and respiratory cycle time (T_Tot_) from patients with stable chronic obstructive pulmonary disease (COPD), recently weaned from mechanical ventilation (MV) and acute respiratory failure (ARF) during the use of nasal high flow (NHF).

In patients with ARF, the average RR of 27 ± 8 min^−1^ during NHF rate of 43 ± 13 L/min was higher compared with the COPD (*P* < 0.001) and successfully weaned patients (*P* < 0.001) and was associated with higher T_I_/T_E_ (0.69 ± 0.12, *P* < 0.05) and shorter T_E_ (1.4 ± 0.38 s, *P* < 0.05) (Table 5). In all patients, the T_Tot_ varied considerably during the recording period (Supplemental Fig. S2, Supplemental Fig. S3, and Supplemental Fig. S4; see https://doi.org/10.6084/m9.figshare.16993108.v1, https://doi.org/10.6084/m9.figshare.16993114.v1, and https://doi.org/10.6084/m9.figshare.16993126.v1). The combined patient data demonstrate the range of breathing patterns that may be seen during the use of NHF therapy (Fig. 2). The data show how the T_I_/T_E_ may tend toward ≤0.5 at the lower RR and toward 1.0 at the higher RR (Supplemental Fig. S5; see https://doi.org/10.6084/m9.figshare.16993129.v3).

**Table 5.** Breathing parameters recorded during nasal high flow therapy in patients with acute respiratory failure

Breathing Parameters	
*n*	9
NHF, L/min	43 ± 12
Respiratory rate, min^−1^	27 ± 8
T_I_, s	0.91 ± 0.23
T_E_, s	1.4 ± 0.38
I:E ratio	0.69 ± 0.12

Data are means ± SD; NHF, nasal high flow; T_I_, inspiratory time; T_E_, expiratory time; I:E, inspiratory to expiratory.

### Positive Airway Pressure during NHF

**Figure 3.**
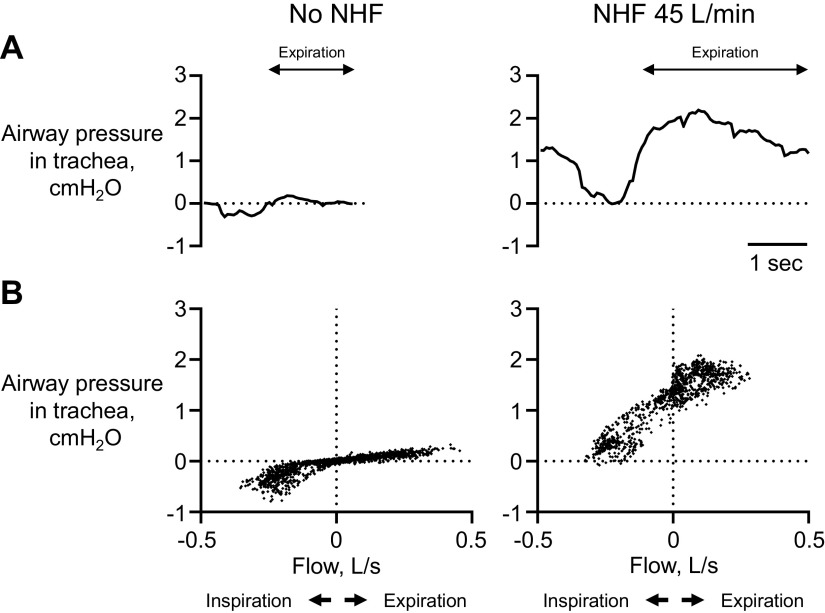
*A*: example recordings of airway pressure (cmH_2_O) measured in the trachea via tracheostomy during no nasal high flow (NHF) and NHF at 45 L/min. *B*: during NHF, dynamic changes in the tracheal pressure during a respiratory cycle are increased due to the resistance-dependent generation of the pressure. The breathing flow was derived from calibrated respiratory inductance plethysmography, and the data are plotted from 1 min of the breathing data from the single patient.

**Figure 4.**
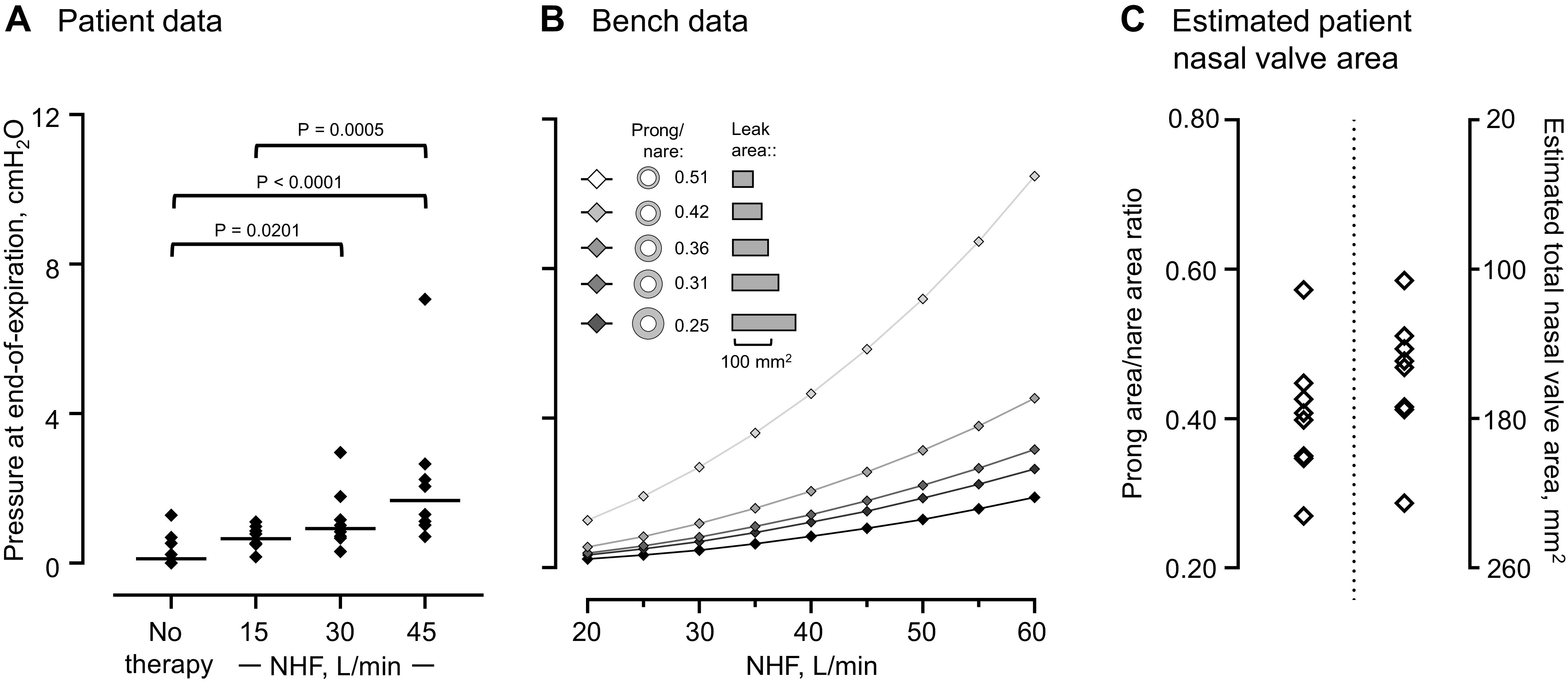
*A*: pressure at the end of expiration measured via a tracheostomy retainer during no nasal high flow (NHF) and then nasal high flow (NHF) is presented. The individual data are shown, and the lines indicate the median value; *n* = 8. *B*: pressure generated by NHF in an upper airway model can be increased with greater NHF rate and occluding more of the nare with the NHF cannula. *C*: the total nasal valve area of the 8 patients was estimated using the bench model data and indicates anatomical variability that may explain between-patient differences in the pressure generated by NHF.

Figure 3 shows example recordings of the airway pressure measured in the trachea of a patient recovering post-MV during no NHF and NHF at 45 L/min. During NHF at 45 L/min, the median pressure at end of expiration, 1.71 cmH_2_O (min: 0.71 and max: 7.06), and peak of expiration, 4.13 cmH_2_O (1.58, 9.81), was higher when compared with NHF at 15 L/min, 0.65 cmH_2_O (0.17 and 1.10), *P* = 0.0003, and 2.03 cmH_2_O (1.12, 4.52), *P* = 0.0117 (Fig. 4*A*). The bench data showed that greater airway pressure is generated when more of the nare is occluded by the prongs and at the higher NHF rate (Fig. 4*B*). The best fit of the relationship between the generated pressure and cross-sectional area from the model nare was applied to the patient data to estimate the nasal valve area; this suggested a high variability of the prong/nare ratios of between 0.27 and 0.57 (Fig. 4*C*).

### Dead-Space Clearance by NHF

**Figure 5.**
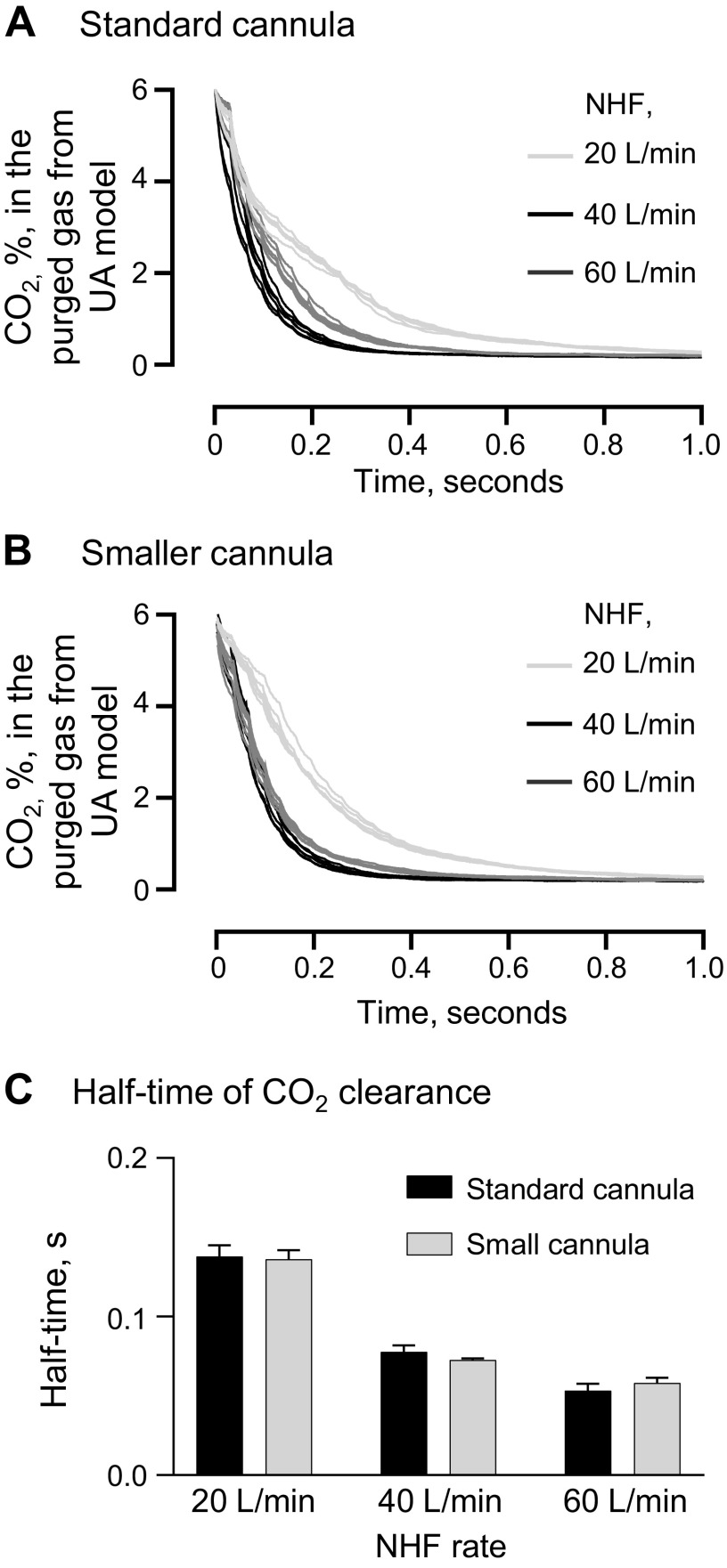
*A* and *B*: recordings of the CO_2_ concentration in the purged gas from an upper airway model during no breathing flow as nasal high flow (NHF) is applied for 1 s, beginning at *time 0*, in a standard-sized nasal cannula (*A*) and smaller sized nasal cannula (*B*). *C*: the time to reduce the concentration of CO_2_ by half in the upper airway (UA) model was increased with rising NHF rates but was not significantly different between the standard-sized cannula and smaller sized one. Data are means ± SD.

The application of NHF resulted in a rapid reduction of CO_2_ in the upper airway model during no breathing using either a standard medium-sized nasal cannula (Fig. 5*A*) or a cannula with reduced diameter (Fig. 5*B*). Increasing the NHF rate significantly reduced the half-time of the CO_2_ clearance and using the smaller cannula did not affect the half-time (Fig. 5*C*).

**Figure 6.**
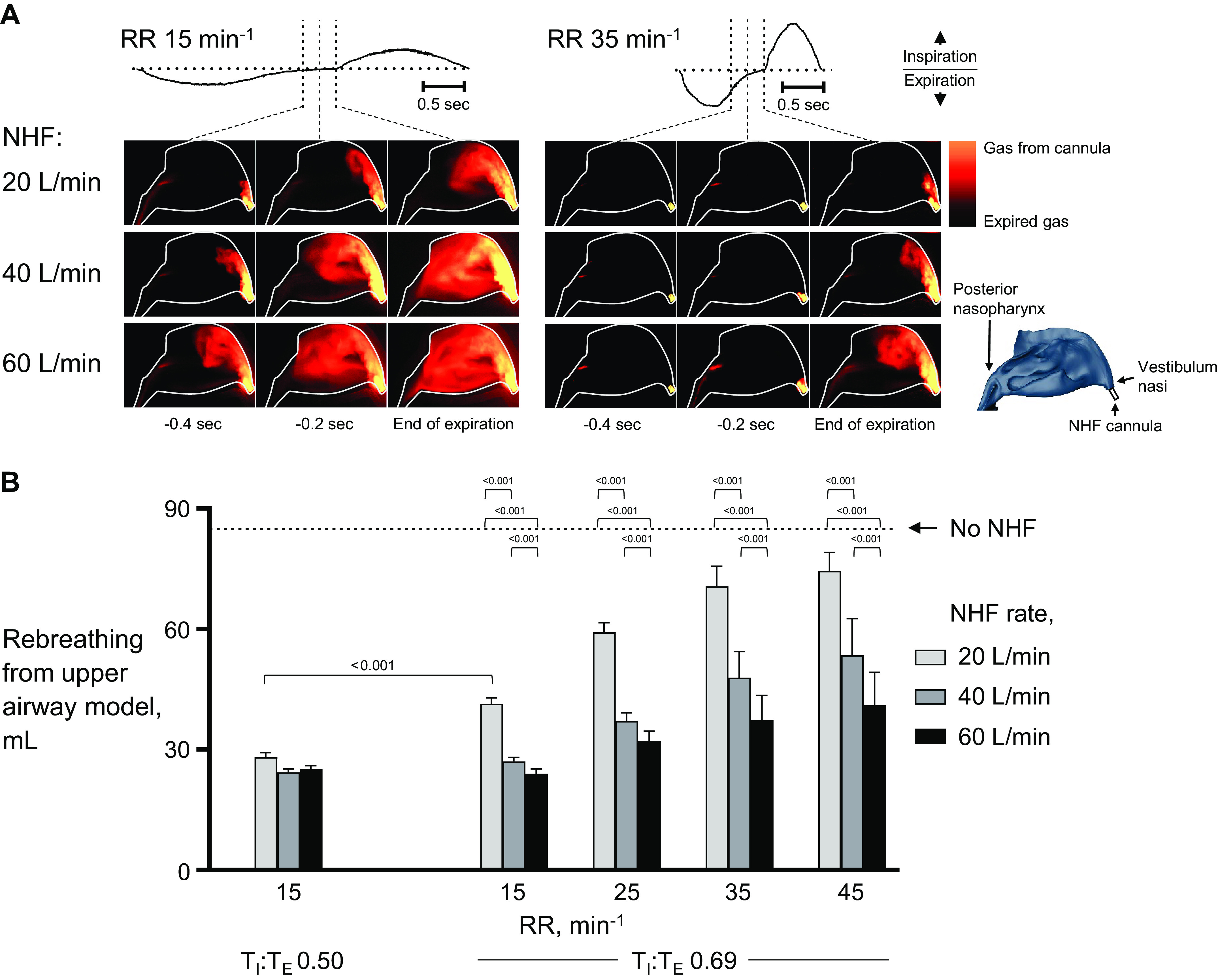
*A*: the images show the gas kinetics in a nasal cavity model (outlined by the solid line) during nasal high flow (NHF) at respiratory rates (RR) of 15 min^−1^ and 35 min^−1^ at the end of expiration. The NHF gas is visualized in yellow-orange as it exits the cannula, as the NHF gas mixes with the expired gas (shown in black) then the color changes to black. During the higher RR, the end-of-expiration period is very short and there is less time for the NHF gas to enter the cavity. *B*: the bar graph demonstrates the volume of expired gas that is rebreathed from the upper airway model during simulated breathing. As the RR is reduced, the rebreathing decreases, which indicates an improvement in the dead-space clearance. T_I_, inspiratory time; T_E_, expiratory time. Data from 20 breaths are presented as means ± SD.

Figure 6*A* demonstrates how NHF dilutes the expired gas in the nasal cavity. Expired gas with elevated CO_2_ concentration continues to fill the nasal cavity throughout the expiration and it is only toward the end of expiration as the flow rate from the lungs reduces that the NHF then dilutes the expired gas. Figure 6*B* demonstrates that as the RR increases, the rebreathing of expired gas rises during NHF. This is partly offset by using a higher NHF rate [during an RR of 45 min^−1^, the cleared volume by NHF at 20 L/min, 40 L/min, and 60 L/min was 9 ± 5 mL, 30 ± 9 mL, and 43 ± 8 mL, respectively, *P* < 0.05 (Fig. 6*B*)]. A reduction of the T_I_/T_E_ to 0.50, from 0.69, during an RR of 15 min^−1^ led to greater cleared volume (56 mL vs. 43 mL, *P* < 0.001). The time to expire the last 100 mL of the tidal volume was negatively correlated with rebreathing (*R*^2^ = 0.89) (Supplemental Fig. S6; see https://doi.org/10.6084/m9.figshare.17303999.v3).

### Modeling of Work of Breathing

**Figure 7.**
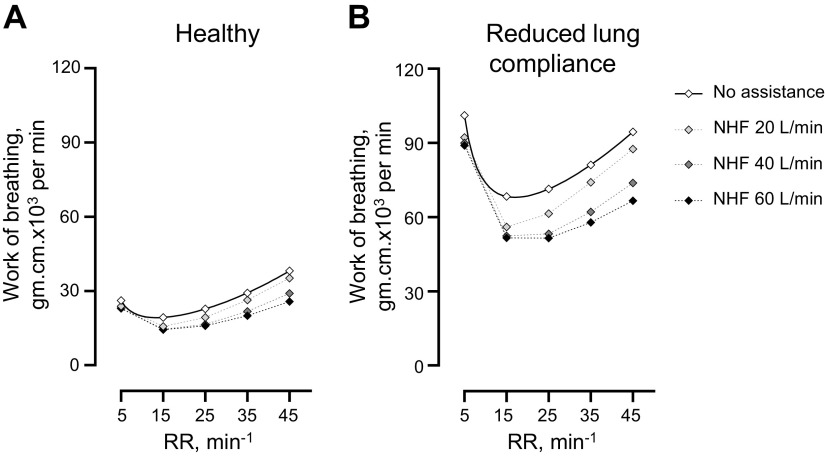
*A*: the model demonstrates the work of breathing may be optimal at respiratory rate (RR) of 15–25 min^−1^ and may rise at the lower RR due to the greater elastic work required for larger tidal volumes and increase at the higher RR due to elevated dead-space ventilation (RR × dead-space volume per breath). The data are based on the work by Otis et al. (18) and modified to show the effect of the dead-space clearance by nasal high flow (NHF) at the rates of 20 L/min, 40 L/min, and 60 L/min; the level of dead-space clearance was determined by the bench experiments of the current study. *B*: a reduced lung compliance requires greater work to maintain the alveolar ventilation and the NHF-mediated reduction in dead-space ventilation may significantly reduce the work of breathing, particularly at the higher RR.

Figure 7 presents the mathematical modeling that shows how the dead-space clearance and subsequent reduction in the dead-space ventilation can decrease the work of breathing. Based on the model, the work of breathing is lowest at the RR between 15–25 min^−1^ and a further decrease in the RR may lead to an increase in the elastic work in order to generate the larger tidal volume that is required to maintain alveolar ventilation (a decrease in the RR from 15 min^−1^ to 5 min^−1^ leads to an increase in the total work by 33% during no NHF and 65%, 72%, and 73% during NHF at 20, 40, and 60 L/min). As the RR rises, the dead-space ventilation becomes greater and can lead to a nonlinear increase in the total work. At the lower RR, the NHF rate does not significantly affect the cleared volume of the dead space and the decrease in the work of breathing is similar between flow rates (−26% during NHF 60 L/min vs. −19% during NHF 20 L/min at RR 15 min^−1^) (Fig. 7*A*). At the higher RR, the higher NHF rates result in a greater volume of dead space that is cleared when compared with the lower NHF rate and therefore a greater decrease in the work of breathing (−32% during NHF 60 L/min vs. −8% during NHF 20 L/min at RR 45 min^−1^) (Fig. 7*A*). In a model of a lower lung compliance, the work of breathing is increased and a reduction in the rebreathing can result in a significant decrease in the work (Fig. 7*B*).

## DISCUSSION

The generated airway pressure measured in the trachea during NHF delivered via a standard medium-size cannula interface was typically lower than usual settings for pressure-controlled therapies. The expiratory pressure was proportional to the flow from the lungs and NHF cannula, and the pressure increased with higher expiratory flow. At the end of expiration when the expiratory flow slows and airway pressure decreases is when the gas from the NHF cannula begins diluting and purging the expired gas from upper airways. In the current study, the ARF patients with a high breathing frequency during NHF presented with very short T_I_ and T_E_, which can suggest a substantially reduced time available for the clearance. The bench data show that when the RR is high the greater NHF rate may be required to reduce the rebreathing, which can lead to an improvement in gas exchange and the work of breathing. In stable patients with lower RR, the T_E_ is greatly increased prolonging the clearance time and efficient clearance may be achieved using the lower NHF rates, which is considered to be more comfortable (19).

### Factors Favoring Dead-Space Clearance

**Figure 8.**
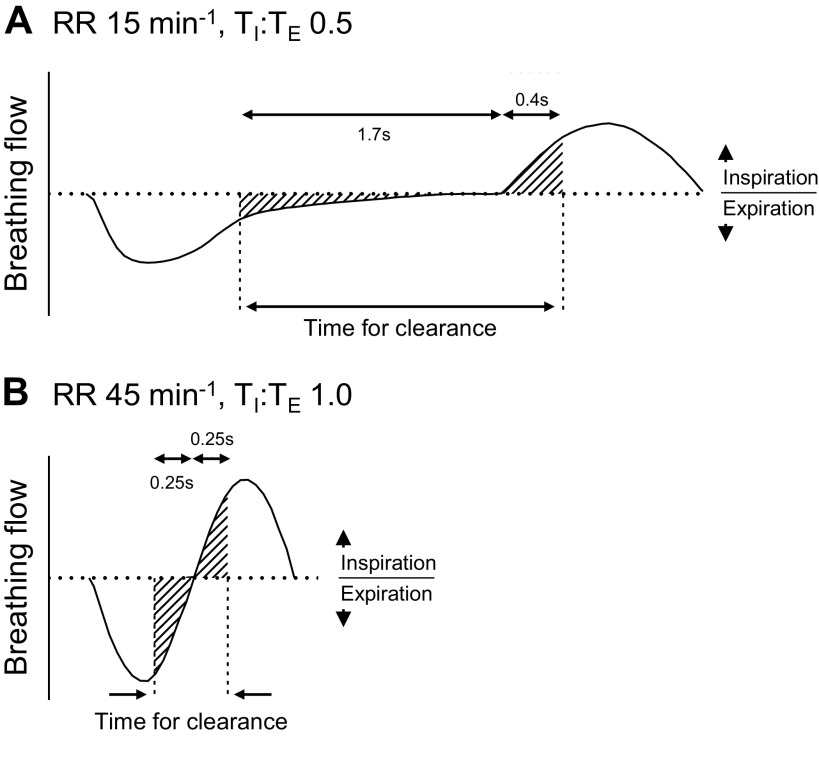
The schematic presents 2 different breathing cycles; a longer breath during an respiratory rate (RR) of 15 min^−1^ and inspiratory time (T_I_):expiratory time (T_E_) of 0.5 with an extended period at the end of expiration (*A*) and a shorter breath during an RR of 45 min^−1^ with a sinusoidal flow pattern (*B*). The shaded areas show the first and last 100 mL of inspiration and expiration, respectively, where most of the dead-space clearance by the NHF may occur. The extended end of expiration period provides more time for the dead-space clearance and maximum clearance may be achieved prior to the start of the inspiration phase. At the higher RR then the breath time is shortened, and there is reduced time for clearance.

The clinical data demonstrate that the breathing cycle can vary considerably over time in the same patient and between patients during NHF therapy. In particular, in ARF patients the RR was observed to be >60 min^−1^ at times and the breathing flow sinusoidal. The bench experiments in the current study show that the clearance of the expired gas from the nasal cavities by NHF occurs predominantly at the end of expiration and in ARF patients with a high RR the time that is available for the clearance may be extremely short. The time that is available for clearance is schematically presented in Fig. 8; when the T_E_ is prolonged, then a longer time is available for the clearance. NHF can rapidly clear the expired gas from the nasal cavities and the complex geometry of the upper airways may limit the maximum dead-space clearance (5, 20, 21). Therefore, in stable patients with lower RR, a lesser NHF rate could be considered that allows efficient clearance and to ensure better comfort (19). However, at the higher RR, the greater NHF rate can increase the clearance due to the very short time that is available to purge the expired gas from the upper airways, consistent with mass balance that includes gas from the cannula and gas from the lungs.

### Impact on Work of Breathing

A decrease in the dead-space ventilation allows for reduced minute ventilation to maintain arterial CO_2,_ which should present as a change in the breathing pattern. NHF is widely reported to decrease the RR that includes ARF patients with a high-breathing frequency of 25–33 breaths/min (2, 9, 14, 22). In ARF the higher RR that may be associated with reduced lung compliance can lead to greater work of breathing required to overcome the increased elastic and viscous resistances (18, 23). Therefore, the dead-space clearance may have a pronounced effect on reducing the work of breathing in these patients by decreasing the required tidal volume and/or RR to maintain the alveolar ventilation (18). A decrease of the RR may be the most effective means to lower the work of breathing through further reductions in dead-space ventilation (24). However, at the lower RR then any further decrease in the breathing frequency could lead to a steep rise in the elastic work, as described by Otis et al. (18) and reducing the tidal volume may be the most effective response to lower the work of breathing (refer to Fig. 7). During sleep, patients with COPD and low baseline RR displayed a reduction in the tidal volume in response to NHF at 20 L/min (7, 25), and is the likely mechanism of the decrease in the work of breathing in these patients (7). However, a reduced compliance of the respiratory system as occurs in ARF may lead to a rapid shallow breathing pattern to lessen the elastic work of breathing and a low RR is unlikely in these patients.

### Positive Airway Pressure during NHF

Positive end-expiratory pressure during ventilation can improve arterial blood oxygenation and protect the lungs (26). NHF is a flow-controlled therapy and the pressure fluctuations during breathing are generated through nonlinear resistance by partial occlusion of the nares and flow from the prongs. Greater occlusion of the nares by the NHF prongs can increase the resistance to flow and lead to higher generated pressure (4). The variability of expiratory pressure measured in the trachea during NHF was associated with differences in the prong/nare ratio and was proportional to the expiratory flow (27). Measuring the pressure in the trachea takes into account the total upper airway resistance and may help to explain the lower positive airway pressure during NHF in the current study when compared with previous data obtained from measurements in the nasopharynx (19, 28, 29). Most resistance to air flow in the upper airways is contributed by the nasal valve, soft palate, and vocal cords, and the internal geometry of each component can independently change leading to variable pressure in the distal airways that is difficult to estimate without direct measurement as performed in the current study.

With NHF, the resistance-dependent generated positive pressure has been associated with extended T_E_ and reduced RR in healthy volunteers (15, 30). This effect may be particularly pronounced in healthy volunteers who expire passively with highly compliant lungs and may help to explain a relatively pronounced decrease of the RR and compensatory increase in tidal volume in response to NHF observed in this population (8, 15, 31). In patients with reduced lung compliance such as in ARF and who may actively expire, the positive expiratory pressure will likely have less effect on the T_E_ and RR; instead, improved gas exchange may explain the reduction of the RR and decrease in the inspiratory effort in response to NHF that is commonly reported in patients with ARF (2, 9, 14, 22). As shown in the current study, the pressure generated by NHF is typically low and it can be hypothesized that an improvement in gas exchange may primarily be due to reduced dead-space ventilation.

### Limitations

Diverse patient populations were represented by small groups to demonstrate the variability and general trend in the T_I_/T_E_ and RR. RIP was used to indirectly measure breathing as NHF is an open system, and the method is considered reliable to time the breathing cycles. In the COPD patient group, NHF at 30 L/min was selected. Rittayamai et al. (32) previously demonstrated that NHF at 30 L/min in patients recovering from a COPD exacerbation with RR of ∼20–25 min^−1^ resulted in the greatest reduction in the inspiratory effort when comparing the flow rates of between 10 L/min and 50 L/min. The current findings would support that the dead-space clearance is efficient at the lower NHF rate when the RR is lower. Increasing the NHF rate can elevate the positive airway pressure but will also raise the noise due to the turbulence of flow from the cannula, which may reduce the compliance in stable patients. During the measurement of pressure in the trachea, the maximum NHF rate was limited to 45 L/min as a result of the intolerance to higher flows by the recently extubated tracheostomized patients. The pressure measurements were performed for a short interval (5 to 10 min), and there was a limited washout period; therefore, the breathing data may not represent patients who receive the therapy for longer.

The study used CO_2_ absorption spectroscopy and volumetric capnography in upper airway models to visualize and quantify the fast-occurring dead-space clearance. Rebreathing can be measured in a person via tracheostomy or by scintigraphy with a radioactive tracer (6); however, the use of a lung simulator avoids the inherent variability that occurs in patients (1, 8) and allowed the authors to control different parts of the respiratory cycle and relate to rebreathing. A standard medium-sized cannula interface was used in the clinical part of the study and is the most frequently selected size. Experiments with a reduced diameter of the prongs showed that this did not affect the half-time of the CO_2_ clearance and that a higher velocity may increase mixing but not the dilution rate due to the limit to maximum dead-space clearance; therefore, the bench-top part of the study was performed using standard medium-sized prongs. The wall air flow source for the NHF was used for the bench-top studies to maintain a steady flow rate during the instantaneous opening of the valve in the static experiments and during different simulated breathing flows. The breathing flow can affect the resistance to the flow that is exiting from the nasal cannula, which may lead to differences in the magnitude of the dead-space clearance and the airway pressure depending on how the NHF is generated and it deserves future investigation.

The bench-top experiments were performed in upper airway models with the mouth closed, and the pressure generated during open mouth may be lower (28). However, as shown previously, the open mouth does not necessarily indicate a leak due to the variable position of the soft palate which can be difficult to determine (10). The unobstructed unidirectional flow that is created by the open mouth in the model will result in highly efficient dead-space clearance, but it may not accurately reflect the clinically observed situation.

### Clinical Significance

The commonly selected size of the NHF interface allows a substantial gap between the prongs and the nares and will tend to result in a lower mean level of airway pressure when compared with typical settings of pressure controlled therapies with a sealed mask; the delivered pressure can be increased with a greater NHF rate and by occluding more of the nare using a larger prong size (3, 4). However, a near complete or full occlusion may result in a steep rise of the airway pressure if the back pressure is not limited, which should be considered when selecting the NHF prong size and the flow source (33). During NHF, the resistance-dependent oscillations of positive airway pressure may affect the breathing through prolonged expiratory time and may lead to a reduction in the RR. Dead-space clearance distinguishes NHF from the use of therapies that require a sealed mask. The clearance of expired gas from the upper airways and the reduction of rebreathing can also lead to a decrease in the RR through the improvement in gas exchange. The model experiments indicate that an increasing RR during NHF would lead to a lower volume of dead space cleared unless a greater NHF rate is used, which can be selected based on the equipment and maximum flow tolerated. The clearance can improve as the breathing becomes slower, and a virtuous cycle may develop between decreasing RR and reduced rebreathing, which may result in improved gas exchange and lower work of breathing. On the contrary, an increasing RR during NHF would lead to less efficient therapy that could suggest a requirement for escalation of therapy. NHF has been shown to reduce the requirement for mechanical ventilation in patients with ARF (34); however, there is a risk of delayed escalation of care, which is associated with higher mortality (35). The respiratory rate oxygenation index was recently introduced to predict the patient outcome during NHF in patients with ARF (36). An elevated RR and a high ${\text{FIO}}_{2}$ lowers the index which predicts a poor prognosis and indicates the importance of monitoring the RR during NHF.

### Conclusions

The breathing frequency substantially affects the efficiency of dead-space clearance by NHF. At the higher RR, a greater NHF rate, apart from increasing the airway pressure, can increase the dead-space clearance and reduce the rebreathing that may eventually lower the respiratory rate and the work of breathing. As the breathing slows, then the lower NHF rates achieve efficient clearance. The study findings suggest that the RR could be an important indicator of not only the respiratory function but also the flow rate selection and therapy efficacy.

## SUPPLEMENTAL DATA

10.6084/m9.figshare.16993051.v3 Supplemental Fig. S1: https://doi.org/10.6084/m9.figshare.16993051.v3.

10.6084/m9.figshare.16993108.v1 Supplemental Fig. S2: https://doi.org/10.6084/m9.figshare.16993108.v1.

10.6084/m9.figshare.16993114.v1 Supplemental Fig. S3: https://doi.org/10.6084/m9.figshare.16993114.v1.

10.6084/m9.figshare.16993126.v1 Supplemental Fig. S4: https://doi.org/10.6084/m9.figshare.16993126.v1.

10.6084/m9.figshare.16993129.v3 Supplemental Fig. S5: https://doi.org/10.6084/m9.figshare.16993129.v3.

10.6084/m9.figshare.17303999.v3 Supplemental Fig. S6: https://doi.org/10.6084/m9.figshare.17303999.v3.

## GRANTS

Fisher & Paykel Healthcare provided equipment and devices for the studies.

## DISCLOSURES

M.P. and S.T. are employees of Fisher & Paykel Healthcare. T.W. and G.N. received research funding from Fisher & Paykel Healthcare.

## AUTHOR CONTRIBUTIONS

M.I.P., T.W., G.N., and S.T. conceived and designed research; M.I.P., U.D., K.-J.F., J.H., M.S., G.N., and S.T. performed experiments; M.I.P., U.D., K.-J.F., J.H., M.S., T.W., G.N., and S.T. analyzed data; M.I.P., T.W., G.N., and S.T. interpreted results of experiments; M.I.P. prepared figures; M.I.P. and S.T. drafted manuscript; M.I.P., G.N., and S.T. edited and revised manuscript; M.I.P., U.D., K.-J.F., J.H., M.S., T.W., G.N., and S.T. approved final version of manuscript.

## References

[1] Delorme M, Bouchard PA, Simon M, Simard S, Lellouche F. Effects of high-flow nasal cannula on the work of breathing in patients recovering from acute respiratory failure. Crit Care Med 45: 1981–1988, 2017. doi:10.1097/CCM.0000000000002693. 28857852

[2] Mauri T, Turrini C, Eronia N, Grasselli G, Volta CA, Bellani G, Pesenti A. Physiologic effects of high-flow nasal cannula in acute hypoxemic respiratory failure. Am J Respir Crit Care Med 195: 1207–1215, 2017. doi:10.1164/rccm.201605-0916OC. 27997805

[3] Braunlich J, Kohler M, Wirtz H. Nasal highflow improves ventilation in patients with COPD. Int J Chron Obstruct Pulmon Dis 11: 1077–1085, 2016. doi:10.2147/COPD.S104616. 27307723 PMC4887061

[4] Pinkham M, Tatkov S. Effect of flow and cannula size on generated pressure during nasal high flow. Crit Care 24: 248, 2020. doi:10.1186/s13054-020-02980-w. 32448344 PMC7245881

[5] Moller W, Celik G, Feng S, Bartenstein P, Meyer G, Oliver E, Schmid O, Tatkov S. Nasal high flow clears anatomical dead space in upper airway models. J Appl Physiol 118: 1525–1532, 2015. doi:10.1152/japplphysiol.00934.2014. 25882385 PMC4482836

[6] Moller W, Feng S, Domanski U, Franke KJ, Celik G, Bartenstein P, Becker S, Meyer G, Schmid O, Eickelberg O, Tatkov S, Nilius G. Nasal high flow reduces dead space. J Appl Physiol 122: 191–197, 2017. doi:10.1152/japplphysiol.00584.2016. 27856714 PMC5283847

[7] Biselli PJ, Kirkness JP, Grote L, Fricke K, Schwartz AR, Smith P, Schneider H. Nasal high-flow therapy reduces work of breathing compared with oxygen during sleep in COPD and smoking controls: a prospective observational study. J Appl Physiol (1985) 122: 82–88, 2017. doi:10.1152/japplphysiol.00279.2016. 27815367 PMC5283854

[8] Delorme M, Bouchard PA, Simon M, Simard S, Lellouche F. Physiologic effects of high-flow nasal cannula in healthy subjects. Respir Care 65: 1346–1354, 2020. doi:10.4187/respcare.07306. 32291309

[9] Jones PG, Kamona S, Doran O, Sawtell F, Wilsher M. Randomized controlled trial of humidified high-flow nasal oxygen for acute respiratory distress in the emergency department: The HOT-ER Study. Respir Care 61: 291–299, 2016. doi:10.4187/respcare.04252. 26577199

[10] Mazmanyan P, Darakchyan M, Pinkham MI, Tatkov S. Mechanisms of nasal high flow therapy in newborns. J Appl Physiol 128: 822–829, 2020. doi:10.1152/japplphysiol.00871.2019. 32078463 PMC7191511

[11] Pisani L, Fasano L, Corcione N, Comellini V, Musti MA, Brandao M, Bottone D, Calderini E, Navalesi P, Nava S. Change in pulmonary mechanics and the effect on breathing pattern of high flow oxygen therapy in stable hypercapnic COPD. Thorax 72: 373–375, 2017. doi:10.1136/thoraxjnl-2016-209673. 28104830

[12] Sztrymf B, Messika J, Bertrand F, Hurel D, Leon R, Dreyfuss D, Ricard JD. Beneficial effects of humidified high flow nasal oxygen in critical care patients: a prospective pilot study. Intensive Care Med 37: 1780–1786, 2011. doi:10.1007/s00134-011-2354-6. 21946925

[13] Sztrymf B, Messika J, Mayot T, Lenglet H, Dreyfuss D, Ricard JD. Impact of high-flow nasal cannula oxygen therapy on intensive care unit patients with acute respiratory failure: a prospective observational study. J Crit Care 27: e9–e13, 2012. doi:10.1016/j.jcrc.2011.07.075. 21958974

[14] Vargas F, Saint-Leger M, Boyer A, Bui NH, Hilbert G. Physiologic effects of high-flow nasal cannula oxygen in critical care subjects. Respir Care 60: 1369–1376, 2015. doi:10.4187/respcare.03814. 25944940

[15] Mundel T, Feng S, Tatkov S, Schneider H. Mechanisms of nasal high flow on ventilation during wakefulness and sleep. J Appl Physiol 114: 1058–1065, 2013. doi:10.1152/japplphysiol.01308.2012. 23412897 PMC3633436

[16] Pinkham M, Burgess R, Mundel T, Tatkov S. Nasal high flow reduces minute ventilation during sleep through a decrease of carbon dioxide rebreathing. J Appl Physiol 126: 863–869, 2019. doi:10.1152/japplphysiol.01063.2018. 30730818

[17] Bruning J, Hildebrandt T, Heppt W, Schmidt N, Lamecker H, Szengel A, Amiridze N, Ramm H, Bindernagel M, Zachow S, Goubergrits L. Characterization of the airflow within an average geometry of the healthy human nasal cavity. Sci Rep 10: 3755, 2020. doi:10.1038/s41598-020-60755-3. 32111935 PMC7048824

[18] Otis AB, Fenn WO, Rahn H. Mechanics of breathing in man. J Appl Physiol 2: 592–607, 1950. doi:10.1152/jappl.1950.2.11.592. 15436363

[19] Narang I, Carberry JC, Butler JE, Gandevia SC, Chiang AKI, Eckert DJ. Physiological responses and perceived comfort to high flow nasal cannula therapy in awake adults: effects of flow magnitude and temperature. J Appl Physiol 131: 1772–1782, 2021. doi:10.1152/japplphysiol.00085.2021. 34709070

[20] Moore CP, Katz IM, Pichelin M, Caillibotte G, Finlay WH, Martin AR. High flow nasal cannula: Influence of gas type and flow rate on airway pressure and CO2 clearance in adult nasal airway replicas. Clin Biomech (Bristol, Avon) 65: 73–80, 2019. doi:10.1016/j.clinbiomech.2019.04.004. 30991233

[21] Onodera Y, Akimoto R, Suzuki H, Okada M, Nakane M, Kawamae K. A high-flow nasal cannula system with relatively low flow effectively washes out CO2 from the anatomical dead space in a sophisticated respiratory model made by a 3D printer. Intensive Care Med Exp 6: 7, 2018. doi:10.1186/s40635-018-0172-7. 29546563 PMC5854566

[22] Mauri T, Alban L, Turrini C, Cambiaghi B, Carlesso E, Taccone P, Bottino N, Lissoni A, Spadaro S, Volta CA, Gattinoni L, Pesenti A, Grasselli G. Optimum support by high-flow nasal cannula in acute hypoxemic respiratory failure: effects of increasing flow rates. Intensive Care Med 43: 1453–1463, 2017. doi:10.1007/s00134-017-4890-1. 28762180

[23] Bernasconi M, Ploysongsang Y, Gottfried SB, Milic-Emili J, Rossi A. Respiratory compliance and resistance in mechanically ventilated patients with acute respiratory failure. Intensive Care Med 14: 547–553, 1988. doi:10.1007/BF00263528. 3065390

[24] Lellouche F, Delorme M, Brochard L. Impact of respiratory rate and dead space in the current era of lung protective mechanical ventilation. Chest 158: 45–47, 2020. doi:10.1016/j.chest.2020.02.033. 32654726

[25] Biselli P, Fricke K, Grote L, Braun AT, Kirkness J, Smith P, Schwartz A, Schneider H. Reductions in dead space ventilation with nasal high flow depend on physiological dead space volume: metabolic hood measurements during sleep in patients with COPD and controls. Eur Respir J 51: 1702251, 2018. doi:10.1183/13993003.02251-2017. 29724917

[26] Dantzker DR, Brook CJ, Dehart P, Lynch JP, Weg JG. Ventilation-perfusion distributions in the adult respiratory distress syndrome. Am Rev Respir Dis 120: 1039–1052, 1979. doi:10.1164/arrd.1979.120.5.1039. 389116

[27] McNamara RM, Cionni DJ. Utility of the peak expiratory flow rate in the differentiation of acute dyspnea. Cardiac vs pulmonary origin. Chest 101: 129–132, 1992. doi:10.1378/chest.101.1.129. 1729059

[28] Parke RL, Eccleston ML, McGuinness SP. The effects of flow on airway pressure during nasal high-flow oxygen therapy. Respir Care 56: 1151–1155, 2011. doi:10.4187/respcare.01106. 21496369

[29] Parke RL, McGuinness SP. Pressures delivered by nasal high flow oxygen during all phases of the respiratory cycle. Respir Care 58: 1621–1624, 2013. doi:10.4187/respcare.02358. 23513246

[30] Davies HW, Haldane JS, Priestley JG. The response to respiratory resistance. J Physiol 53: 60–69, 1919. doi:10.1113/jphysiol.1919.sp001859. 16993440 PMC1405605

[31] Parke RL, Bloch A, McGuinness SP. Effect of very-high-flow nasal therapy on airway pressure and end-expiratory lung impedance in healthy volunteers. Respir Care 60: 1397–1403, 2015. doi:10.4187/respcare.04028. 26329355

[32] Rittayamai N, Phuangchoei P, Tscheikuna J, Praphruetkit N, Brochard L. Effects of high-flow nasal cannula and non-invasive ventilation on inspiratory effort in hypercapnic patients with chronic obstructive pulmonary disease: a preliminary study. Ann Intensive Care 9: 122, 2019. doi:10.1186/s13613-019-0597-5. 31641959 PMC6805835

[33] Locke RG, Wolfson MR, Shaffer TH, Rubenstein SD, Greenspan JS. Inadvertent administration of positive end-distending pressure during nasal cannula flow. Pediatrics 91: 135–138, 1993. doi:10.1542/peds.91.1.135. 8416477

[34] Rochwerg B, Granton D, Wang DX, Helviz Y, Einav S, Frat JP, Mekontso-Dessap A, Schreiber A, Azoulay E, Mercat A, Demoule A, Lemiale V, Pesenti A, Riviello ED, Mauri T, Mancebo J, Brochard L, Burns K. High flow nasal cannula compared with conventional oxygen therapy for acute hypoxemic respiratory failure: a systematic review and meta-analysis. Intensive Care Med 45: 563–572, 2019. doi:10.1007/s00134-019-05590-5. 30888444

[35] Kang BJ, Koh Y, Lim CM, Huh JW, Baek S, Han M, Seo HS, Suh HJ, Seo GJ, Kim EY, Hong SB. Failure of high-flow nasal cannula therapy may delay intubation and increase mortality. Intensive Care Med 41: 623–632, 2015. doi:10.1007/s00134-015-3693-5. 25691263

[36] Roca O, Caralt B, Messika J, Samper M, Sztrymf B, Hernandez G, Garcia-de-Acilu M, Frat JP, Masclans JR, Ricard JD. An index combining respiratory rate and oxygenation to predict outcome of nasal high-flow therapy. Am J Respir Crit Care Med 199: 1368–1376, 2019. doi:10.1164/rccm.201803-0589OC. 30576221

